# Recent Discoveries on Marine Organism Immunomodulatory Activities

**DOI:** 10.3390/md20070422

**Published:** 2022-06-27

**Authors:** Eleonora Montuori, Donatella de Pascale, Chiara Lauritano

**Affiliations:** 1 Department of Chemical, Biological, Pharmaceutical and Environmental Sciences, University of Messina, Viale F. Stagno d’Alcontres 31, 98166 Messina, Italy; eleonora.montuori@szn.it; 2 Ecosustainable Marine Biotechnology Department, Stazione Zoologica Anton Dohrn, Via Acton 55, 80133 Naples, Italy; donatella.depascale@szn.it

**Keywords:** marine organisms, immunomodulatory activity, inflammation, marine drugs, cancer

## Abstract

Marine organisms have been shown to be a valuable source for biologically active compounds for the prevention and treatment of cancer, inflammation, immune system diseases, and other pathologies. The advantage of studying organisms collected in the marine environment lies in their great biodiversity and in the variety of chemical structures of marine natural products. Various studies have focused on marine organism compounds with potential pharmaceutical applications, for instance, as immunomodulators, to treat cancer and immune-mediated diseases. Modulation of the immune system is defined as any change in the immune response that can result in the induction, expression, amplification, or inhibition of any phase of the immune response. Studies very often focus on the effects of marine-derived compounds on macrophages, as well as lymphocytes, by analyzing the release of mediators (cytokines) by using the immunological assay enzyme-linked immunosorbent assay (ELISA), Western blot, immunofluorescence, and real-time PCR. The main sources are fungi, bacteria, microalgae, macroalgae, sponges, mollusks, corals, and fishes. This review is focused on the marine-derived molecules discovered in the last three years as potential immunomodulatory drugs.

## 1. Introduction

The ocean covers seventy percent of the planet’s surface [1] and is characterized by a huge biodiversity in term of species and the natural products that they may produce. It has recently been reported that species living in the world’s oceans range from 700,000 to one million, but, up to date, the species studied for their possible bioactivities represent only a small percentage of the totality of existing marine organisms [2,3]. Marine species have been shown to produce a plethora of compounds with communication and defensive roles in the natural environment and potential bioactivities for human health. Given the physical and chemical properties of the marine environment, almost all classes of organisms that inhabit it present a diversity of molecules with unique structural characteristics [2]. The biodiversity of marine-derived products is significantly higher than compounds of terrestrial origin [4]. This has attracted the attention of several researchers, as well as companies, interested in the prevention and treatment of human pathologies. Marine-derived compounds are currently used in the food, cosmetic, pharmaceutical, and aquaculture sectors [5]. Recent advances have been reported for biomaterial and bioenergy applications, as well [4]. The search for new and non-toxic compounds from natural sources for cancer treatments is necessary, due to, sometimes, the low specificity of the mechanisms of action of chemotherapy and radiotherapy, as well as side the effects in patients. Over the past decade, both innate and adaptive immune-stimulating compounds have been used to prevent and treat various diseases, including cancer [6].

Dysfunctions of the immune system lead to the development of autoimmune disorders, allergies, chronic inflammation, and cancer. About 7.6–9.4% of the world’s population is affected by diseases that affect the immune system [7]. Several drug research and developmental programs worldwide are focused on searching for bioactive compounds obtained from natural sources [8]. So far, 9 of the 14 commercially available marine-derived drugs are used for cancer treatment, and many more are in clinical trials [9].

**Figure 1:**
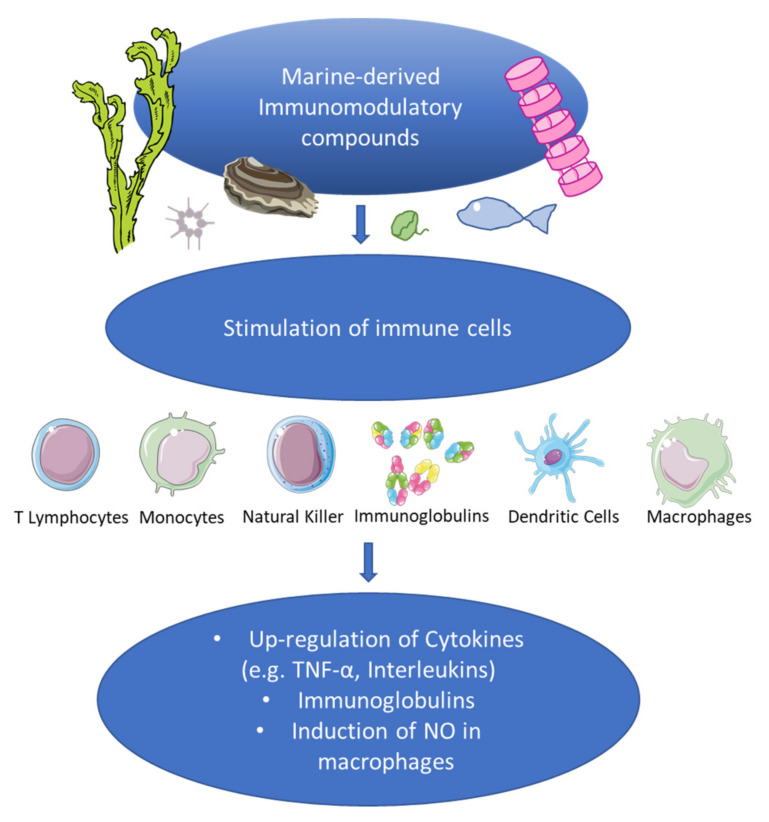
Immunomodulatory effects of marine-derived compounds. TNFα stands for tumor necrosis factor alpha, IL-1 for interleukin-1, IL-6 for interleukin-6, and NO for nitric oxide.

Immunomodulation includes processes aimed at modifying and/or regulating the immune response for therapeutic purposes. Immunomodulators are substances that are used to produce effects on the immune system and are basically divided into immunosuppressants and immunostimulants. Currently, global epidemiological data indicate an increase in immunological diseases, from an estimated prevalence of 3.2% between 1965 and 1995 to 19.1% ± 43.1 reported in 2018 [10,11]. By 2026, it is estimated that the size of the global autoimmune-disease diagnosis market, which is currently worth 4.1 billion dollars, will reach 6.3 billion dollars [12]. This has stimulated the search for a class of molecules, generally immunomodulatory, that is capable of increasing or suppressing the immune response in immune-mediated diseases [13,14]. The body’s first line of defense is innate immunity, which acts rapidly after an invading pathogen. The cells that act during this phase of the immune response are natural killer lymphocytes, neutrophils, and macrophages. The latter are one of the most important classes of antigen-presenting cells (APCs) that play roles in innate immunity and regulate adaptive immunity. Cytokines secreted by activated macrophages cause inflammatory responses and are essential for host defenses against invading pathogens [15] and tumor cells, recruiting and activating other cells at the site of infection. In response to this stimulus, macrophages increase the production of reactive nitrogen intermediates such as nitric oxide (NO); reactive oxygen species (ROS); and pro-inflammatory cytokines such as tumor necrosis factor alpha (TNF-α) and interleukin 6 (IL-6) [16]. However, uncontrolled and prolonged inflammatory responses are harmful to the host and promote the pathogenesis of many inflammatory diseases, such as metabolic disorders [17]. Therefore, macrophages have often been used to evaluate the immunomodulatory effects of bioactive compounds of natural origin, and studies have been conducted by evaluating the expression levels of cytokines such as TNF-α, interleukins such as IL-1 and IL-6, and induction of NO in macrophages (Figure 1).

During inflammatory diseases, such as autoimmune diseases and sepsis, immunosuppressive molecules can serve as therapeutic agents [18]. Likewise, molecules that participate in immune activation can induce immune responses against cancer and infectious diseases. Recently, there has been an increased interest by the scientific community in searching compounds that are able to modulate the immune response since these compounds have potential applications in the fields of immunopharmacology and oncotherapy [19,20]. According to a press release of MarketWatch (https://www.marketwatch.com/press-release/marine-derived-drugs-market-size-2022-global-status-report-industry-valuation-primary-and-secondary-research-detailed-study-growth-orientation-statistics-and-forecast-2028-2022-03-30; accessed on 21 June 2022), the global marine-derived-drugs market is expected to exceed more than US $2516.8 million by 2024 at a CAGR (Compounded Average Growth Rate) of 9.1%. Studies very often focus on the effects of marine-derived compounds on macrophages, as well as lymphocytes, by analyzing the release of mediators (cytokines) by using the immune assay enzyme-linked immunosorbent assay (ELISA), Western blot, immunofluorescence, and real-time PCR. By only using the PCR, researchers may have an idea of the expression of a certain transcript, but they do not know if the transcript is translated into protein. Hence, it is very important to use a combination of techniques in order to better clarify the immunomodulatory mechanism of action. Several papers have been published on immunomodulatory compounds from the sea and many research activities/projects performed to isolate new immunomodulatory activities. Two reviews are available in this field [6,21], both published in 2019; one was only focused on marine microalgae [6], while the other included also other marine species [21]. In light of the attention of the scientific community on the topic, many other papers have come out after the publication of these reviews, and the aim of the current review is to summarize recent findings on marine organisms’ immunomodulatory activities, reporting results not only of pure molecules but also of promising raw extracts and fractions. Finally, our review discusses new trends and scientific directions. 

## 2. Compounds Available on the Market

**Figure 2:**
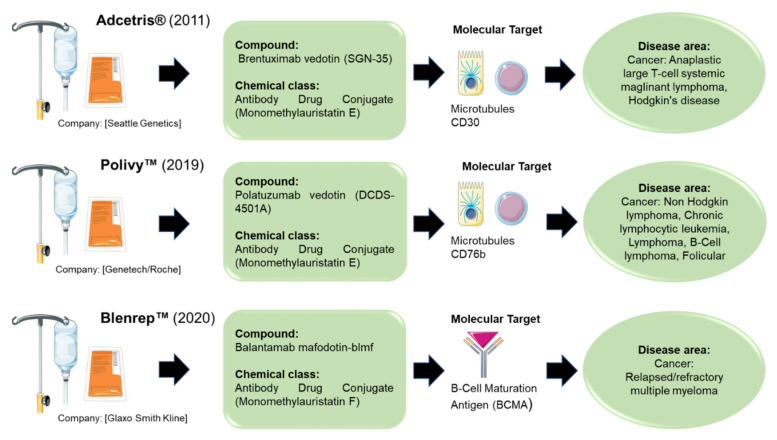
The figure shows the compounds which target the immune system approved by the Food and Drug Administration (FDA) for marketing (https://www.midwestern.edu/departments/marinepharmacology/clinical-pipeline; accessed on 5 May 2022).

There are three marine-derived compounds on the market known to target/modulate the immune system (Figure 2). In particular, in 2011, Adcetris^®^ was approved by the food and drug administration (FDA) and marketed by Seattle Genetics. It is antibody–drug conjugate CD30-directed and indicated for the treatment of patients with Hodgkin lymphoma (after the failure of autologous stem cell transplantation or at least two prior multi-agent chemotherapy regimens), as well as for patients with systemic anaplastic large-cell lymphoma (after the failure of at least one prior chemotherapy regimens). After the discovery of dolastatin 10, with potent antitumor activity, firstly isolated from the sea hare *Dolabella auricularia*, and then from the marine cyanobacterium *Symploca* sp. VP642 [22], various synthetic analogues, named “auristatins”, have been produced and studied. One of these auristatins, named monomethyl auristatin E, became part of the new drug brentuximab vedotin, an antibody–drug conjugate [23,24,25].

In the 2019, the FDA approved Polivy™, marketed by Genetech/Roche. The compound is Polatuzumab vedotin (DCDS-4501A), an antibody–drug conjugate that is CD79b-directed [26] and has a B-receptor component; it is indicated for adult patients with relapsed or refractory diffuse large-B-cell lymphoma. It was isolated from mollusk/cyanobacteria [27]. 

Recently, in 2020, the FDA approved Blenrep™, which is marketed by GlaxoSmithKline (GSK). The compound at the base of this drug is named Belantamab madofotin-blmf, an antibody–drug conjugate directed against the B-cell maturation antigen (BCMA); it is indicated for adult patients with multiple myeloma. It was isolated from mollusk/cyanobacteria [28].

## 3. Marine Microorganisms as Source of Immunomodulatory Compounds

### 3.1. Fungi and Bacteria

Fungi are often reported for their production of anti-inflammatory compounds [29]. Some fungi are known to be able to stimulate the immune system, have anticancer activity, and rejuvenate the immune system weakened by chemotherapy and radiotherapy, and, for these reasons, they have been considered excellent candidates for immunotherapy [18]. There are already many compounds purified from different species of marine fungi that have been shown to affect the immune system, such as semi-vioxanthin [30] and azonazine [31], showing immunomodulatory and anti-inflammatory activities tested on a RAW264.7 cells, respectively. Some studies have also shown that fungi are a source of polysaccharides, compounds that are increasingly appreciated for the treatment of immune diseases and for their potential probiotic properties [32].

The α-D-glucan YCP was purified from the mycelium of the mushroom *Phoma herbarum* YS4108, which showed significant immunomodulatory functions by regulating T lymphocytes and dendritic cells in vitro, activating macrophages in vitro, and increasing the phagocytotic activity in vitro and in vivo [33,34]. In a recent study by Wei Liu et al., the α-D-glucan YCP was administered to mice suffering from dextran sodium sulfate (DSS)-induced acute colitis. After seven days, the YCP proved effective in relieving the clinical symptoms of mice with colitis, the restoration of intestinal immune homeostasis, and the remission of mucosal damage. Furthermore, the YCP blocked the overexpression of the pro-inflammatory cytokines IL-1β, IL-6, and TNF-α induced by DSS in the colon. The levels of IL-10 and IL-22 in the tissues were also significantly increased, resulting in the regeneration of damaged tissues. YCP caused important alterations on the specific microbiota, including Firmicutes, Bacteroidetes, Proteobacteria, Clostridiales, and Lachnospiraceae, which are closely related to immune regulation and mucus repair [35]. In summary, the α-D-glucan YCP from the marine fungi *Phoma herbarum* YS4108 may be a candidate for the treatment of ulcerative colitis [34]. 

Marchese et al. [36] applied a high-throughput drug-screening technology for the bio-prospect of a large library of Irish deep-sea organism extracts to induce human mesenchymal stem cell (hMSC) differentiation. The library also included filamentous fungi, which showed a 6.8% success rate in reducing inflammation of activated macrophages. The extracts, in fact, have shown a significant reduction of the production of pro-inflammatory cytokines, such as TNFα and IL-1β, representing valid candidates for the discovery of anti-inflammatory drugs [36].

The excessive use of antibiotics, antiseptics, and chemotherapy agents has important environmental implications and has led to the development of multidrug-resistant bacteria [37]. The accumulation of chemical and antibiotic residues in the environment is compromising the natural balance of flora and fauna [38,39,40]. Probiotic bacterial species have been shown to possess antimicrobial capabilities against various pathogens, such as the new marine bacterial strain isolated by Wasana et al., *Pseudoalteromonas xiamenensis*, which has been shown to be a potential probiotic candidate to stimulate the host’s immune system to combat disease and to improve environmental suitability, phenomena evaluated by using murine macrophages RAW264.7 [41]. According to the definition of the World Health Organization, probiotics are living microorganisms which, when administered in adequate quantities, confer health benefits [42] such as improvement of immunity [43], disease control [44,45], tolerance to stress, improved digestion, and improved absorption of food [44].

### 3.2. Microalgae

Algae, and, in particular, microalgae, are one of the most promising sources of natural compounds that can be used as substitutes for common synthetic drugs. What especially makes them promising candidates for the discovery of immunomodulating substances to be used as new drugs is their long evolutionary and adaptive diversification to a multitude of habitats and extreme conditions [46], as well as easy sampling and cultivation with respect to macroorganisms [6]. In addition, many microalgae have been observed to have immunomodulatory effects in human and mouse models [47]. In particular, it has been repeatedly shown that polysaccharides extracted from marine microalgae have powerful activities on the immune system. 

Polysaccharides have attracted particular attention because they are highly bioactive compounds which generally show no toxicity [16]. Marine microorganisms, in particular, microalgae, have been shown to be excellent eco-sustainable producer of polysaccharides [48]. Costa et al. [48] have also reported that polysaccharide immunomodulatory activities were related to the molecular weight of the polysaccharides. For example, it was shown that low-molecular-weight exopolysaccharides, extracellular polysaccharides, from the microalga *Porphyridium cruentum* had better immunomodulatory effects. Exopolysaccharides are secreted by the microalgae in the water medium [49]. Mutmainnah et al. [49] reported a study on the growth of the microalga *Porphyridium cruentum* and the production of exopolysaccharides by Fourier-transform infrared spectroscopy (FTIR). They showed the composition of exopolysaccharides consisting of dominant bonds, called phenolic bonds and polysaccharide bonds [49]. Very recently, Risjani et al. showed that exopolysaccharides from *P. cruentum* could stimulate the immune system of the Pacific shrimp *Litopenaeus vannamei* in response to vibriosis caused by *Vibrio harveyi* [50]. In fact, they observed an increase in the total value of hemocytes (THC) and phagocytotic activity (PA).

Some marine microalgae, such as *Haematococcus pluvialis*, *Spirulina plantesis* (*Arthospira plantesis*), *Dunaliella salina*, and *Chlorella* sp., are cultivated for the production of pigments to be used for food supplements and natural food coloring [51,52]. Microalgal pigments have attracted the market of functional food for their anti-inflammatory and antioxidant activities [53,54,55]; in fact, they are widely utilized [52]. The major classes of pigments found in microalgae are phycobilin, carotenoids, and chlorophylls [52].

A 2022 in silico study that used molecular docking [56] showed that microalgae pigments β-carotene, phycocyanobilin, astaxantin, 9-cis-β-carotene, and violaxantin docked pro-inflammatory proteins such as TNFα, IL-6, and NF-κB-inducing kinase (NIK). The aims of the researchers were (1) to investigate the immunomodulating activity of microalgal pigments and (2) to propose a first clue on the mechanism of microalgal pigments modulating the human immune system. The binding between the pigments and protein is mostly attributed to the Van der Waals interaction. Thus, these interactions suggest the interaction of the protein with its receptor. The lower the binding energy, the better is the binding of ligand and protein [57]. Researchers found that β-carotene has the lowest binding energy to IL-6, with a binding energy of −7.9 Kcal/mol. The IL-6 dysregulation could promote chronic inflammation and autoimmunity [58,59]. Therefore, the exploration and development of a compound that is capable of inhibiting and binding IL-6 could be useful for treating autoimmune disease and preventing cytokine cascade. The 9-cis-β-carotene has the lowest binding energy to TNF-α, with a binding energy of −7.9 Kcal/mol. TNF-α signaling activates the NF-kB pathway and mitogen-activated pathway kinase (MAPK) cascade. To start the TNF signaling, TNF-α can bind to TNF receptor (TNFR) type 1 or TNFR type 2. TNFR1 acts as a pro-inflammatory factor and promotes apoptosis. Thus, it could be useful in alleviating the cytokine storm in autoimmune disease by blocking the attachment of TNF-α to TNFR1 [60]. The phycocyanobilin has the lowest binding energy to NF-κB-inducing kinase (NIK), with a binding energy of −9.9 Kcal/mol. NIK regulates the NF-kB pathway and supports the TNF-α signaling cascade. The exploration of natural compounds that are able to inhibit NIK can lead to the discovery of compounds that are capable of inhibiting the NF-kB pathway and modulating the immune system [61]. Similar to this, microalgae pigment may support the immune system modulation and prevent and attenuate chronic inflammatory [56].

The effects of β-glucans extracted from the microalga *Phaeodactylum tricornutum* have been recently evaluated following their application as food supplements for seabream *Sparus aurata* juveniles, a valuable fish species for European aquaculture [62]. *P. tricornutum* is a marine diatom that is rich in numerous health-beneficial compounds, including β-glucans [63,64,65]. These polysaccharides can act as prebiotics, promoting the growth of the commensal microbiota and directly stimulating the innate immune system through interaction with specific cell receptors [66]. It has been noted that those with higher biological activity exhibit a common pattern, namely a repeating chain of β-D-glucopyranosyl units linked to (l-3), with single branched β-D-glucopyranosyl units randomly attached by 1–6 bonds or 1–4 [67,68]. These repeating patterns, called pathogen-associated microbial patterns (PAMPs), are a common feature with bacterial lipopolysaccharides (LPS) and can be recognized by host-cell pattern-recognition receptors (PRRs). Upon recognition, they can elicit an inflammatory response and activate the host’s innate immune cells [61]. Intensive fish production increases the risk of infections caused by opportunistic bacteria, thus creating a condition that negatively affects immune function [69]. Recently, young sea bream *Sparus aurata* were fed for two weeks with β-glucans derived from microalgae, resulting in an increase in immune parameters and resistance to pathogens [70], as already reported in other studies where β-glucans were administered orally [70,71,72]. In a review published in 2022, Hwang et al. reported the chemical composition of algal polysaccharides (alginate, fucoidan, ascophyllan, and porphyrin) and their applications in the treatment of cancer, infectious diseases, and inflammation [73]. Most of these microalgae-derived compounds act as a vaccine adjuvant by enhancing the immune response by activating APCs [2,74,75]. Overall, the number of microalgal species that produce immunomodulating molecules has not yet been adequately characterized and requires further investigations. The most recent immunomodulatory chemical constituents isolated from marine microorganisms mentioned in the current review are listed in Table 1. 

## 4. Macroorganisms as Source of Immunomodulatory Compounds

### 4.1. Macroalgae

Macroalgae are a source of many compounds whose beneficial activity on human body has been widely demonstrated [76]. It has been shown, for instance, that they contain about 60 distinct elements, including calcium, phosphorus, sodium, magnesium, iron, copper, manganese, potassium, vanadium, and iodine, and have favorable nutritional values thanks to the high content of carotenoids, proteins, dietary fibers, essential fatty acids, vitamins (vitamins C, D, E, K, and B complex), and minerals [76]. In fact, in many parts of the world, algae are an integral part of the diet—for instance, in Asian countries [77]. In Southeast Asia, seaweeds have been used for a long time as a food source and a component of traditional medicine preparations. The earliest records of the use of algae as a food source for humans dates back to the fourth century in Japan and the sixth century in China [77]. Since then, seaweed has become a food source that constitutes up to 25% of the human diet in countries such as Japan, China, and South Korea. In addition, regions in North and South America [78] have also increased their consumption of algae even further, as well as in Europe, mainly in France, Italy, Greece, and Ireland [79]. Many algal extracts have been shown to have activities that stimulate the immune system. 

Among the compounds derived from algae, ulvan, a gelling sulfated polysaccharide extracted from *Ulva Ohnoi*, was found to possess immunomodulatory activity. Ulvan was depolymerized in the different fractions, namely U7, U9, U13, U21, and U209, where 7, 9, 13, 21, and 209 correspond to their molecular weights expressed in kDa. Researchers tested the ability of ulvan to alter the inflammatory response in vitro in murine RAW 264.7 macrophages stimulated by lipopolysaccharide (LPS). Fractions did not show cytotoxicity when tested at concentrations below 100 µg/mL for 48 h. Regarding the immunomodulatory activity, assessed by analyzing the expression of inflammatory mediators, it was observed that fractions with the highest molecular weights (U21 and U209) showed an immune response at 100 µg/mL, leading to an increase in IL-1β, IL-6, and IL-10; improvement of LPS-induced inflammation; and a decrease in prostaglandin E2 (known as a potent inflammatory mediator generated by cyclooxygenase 2 conversion of arachidonic acid [80]). From the chemical characterization of the compound, it was seen that the two main sugar constituents are rhamnose and glucuronic acid. It is known that cutaneous fibroblasts and keratinocytes are capable of directly recognizing these two sugars [81], thus making ulvan a promising compound for the production of drugs for ectopic use [82].

In recent years, it has also been seen that brown algae are a source of biologically active compounds such as fucoidans, which exhibit immunostimulant effects by activating various immune cells, as well as antioxidant, antitumor, antiviral, anti-allergic, and anticoagulant effects [83,84,85]. Fucoidans are polysaccharides that consist of L-fucose and sulfate ester groups [86]. Many in vivo and in vitro studies have investigated the immunomodulatory properties of fucoidans extracted from brown seaweed [87,88,89,90]. Recently, the effects of fucoidans extracted from four species of brown algae on human peripheral blood dendritic cells (DCs) have been compared. Fucoidans were extracted from *Ecklonia cava*, *Macrocystis pyrifera*, *Undaria pinnatifida*, and *Fucus vesiculosus.* The fucoidans extracted from *Ecklonia cava* showed greater pro-proliferative activity in T cells and induced an increase in the production of INF-γ [91].

*Caulerpa cupressoides* sulphated polysaccharides (SPs) have been shown to represent potential candidates for the development of new products with immunomodulatory properties of biomedical interest, with applications including the treatment of hypo-immunity or immunodeficiency conditions. Green algae SPs consist mainly of galactose, xylose, arabinose, mannose, rhamnose, glucuronic acid, and/or glucose [92]. In the study by Barbosa et al., two galactans, sulfates and pyruvates (named SP1 and SP2), were obtained from a SP-rich fraction from the alga *C. cupressoides*. Both galactans have similar structures, differing only for the presence of a sulfation in position four in one of the SP2 units. They had an immunostimulant effect that was evidenced by the increased production of NO, endoplasmic reticulum oxidoreductin (ERO), and of the cytokines IL-6 and TNF-α in murine macrophages RAW 264.7. These results indicated that C-4 sulfation is not essential for the immunomodulatory action of these galactans [13].

Another very recent study by Liu et al. highlighted that a sulphated oligosaccharide derived from *Gracilaria lemaneiformis* (GLSO) had an immunomodulatory effect in the responses of Th1 lymphocytes by limiting the activation of T cells in mice immunized with ovalbumin. In vitro, GLSO was able to inhibit the activation system of OVA-specific CD4+ T cells, and, in vivo, it was able to inhibit the production of INF-γ by T cells in mice immunized by ovalbumin [93].

### 4.2. Sponges

Sponges represent an abundant reserve of compounds with immunomodulatory, anti-inflammatory, and anticancer activities of pharmaceutical importance [94]. Several drug-discovery and -development programs are focused on searching for bioactive compounds from marine sponges [95]. The first marine-derived drug on the market was commercialized as Cytosar^®^ by the company Pfizer. Cytosar-U^®^ was approved by the Food and Drug Administration (FDA) in 1969 for the treatment of leukemia and lymphoma. The active compound, cytarabine, or Ara-C, is a synthetic analog of a C-nucleoside isolated from the Caribbean sponge *Tethya crypta* and can inhibit the DNA polymerase as a mechanism of action. Another compound isolated from a sponge and actually on the market is Vira-A^®^, which was commercialized by Mochida Pharmaceutical Co.; it was the first antiviral (against herpes simplex virus) marine drug approved by the FDA in 1976 [96]. The active compound is vidarabine, or Ara-A, which was originally isolated from the sponge *T. crypta* and is active by inhibiting viral DNA polymerase. Other compounds were then used to develop cancer drugs such as Avarol, an hydroquinone sesquiterpenoid that was isolated from *Dysidea avara* [97]; colon cancer cells showed sensitivity to this compound (IC50 < 7 mM) [98].

Halichondrin-B was isolated from *Halichondria okadai*, *Axinella* sp., and *Phakellia* sp. [99,100]. The simplified analogue of Halichondrin-B, Halaven^®^, is a chemotherapy drug that is used against metastatic breast cancer and is available on the market from Eisai S.r.l. [101]. In 2000, a study conducted on *Dysidea* sp. showed that the extracted polyoxygenated sterols have a strong selective immunosuppressive capacity, as they block the interaction between IL-8 and its receptor [102]. Moreover, two other compounds, Pateamina A isolated from *Mycola* sp. and Discodermolide isolated from *Discodermia dissoluta*, were able to inhibit IL-2 production in T and B lymphocytes, displaying unique immunosuppressive and cytotoxic properties [21]. 

In a recent study by Gunathilake et al. [103], the researchers tested in vivo and ex vivo the crude extract of a new Sri Lankan sponge *Haliclona* (*Soestella*) sp. by administering it to albino Wistar mice for a period of 14 consecutive days, at various concentrations: 15, 10, and 5 mg/kg. The experiments showed immunomodulatory activity in mice that were administered with the highest concentration of *Haliclona* (*Soestella*) sp. crude extract (HSCE). Compared to the control, the extract showed a decrease in immune cells (white blood cell, lymphocytes, platelets, mesenchymal cells from bone marrow, and splenocytes) and in the splenocytic index, while there was an increase in the neutrophil:lymphocyte ratio. The immunomodulatory activity was confirmed by the increase in plasma levels of TNF-α in mice treated with 15 mg/kg of HSCE [103]. 

Last year, another study showed the isolation of two classes of marine natural products, agelasine diterpenoids and ageliferins, from the organic extract of the demosponge *Astrosclera willeyana*. The ageliferines compounds were able to inhibit ubiquitin-protein ligase (E3), called Cbl-b. The ubiquitin Cbl-b is essential for the negative regulation of T cell activation and reduces the immune response of cancer cells [104]. The authors evaluated Cbl-b ubiquitin ligase inhibition using the Cbl-b biochemical assay. Of these metabolites, Ageliferins were the most potent in regard to inhibiting Cbl-b, with IC50 values ranging from 18 to 35 µM, compared to ageliferine diterpenoids, with IC50 > 50 µM [105].

### 4.3. Other Species 

In recent years, various studies have been conducted on the discovery and purification of compounds deriving from marine species that show biological and, in particular, immunomodulatory activity. Among the most studied active animals in recent years are bivalve mollusks, fishes, corals, and other marine animals [106]. Often, some of these marine animals are a source of food, and it has often been shown that some extracted peptides have immunomodulatory and anti-inflammatory activity. Many food-borne peptides are known to have regulatory effects on immune responses [106,107]. Thanks to studies on marine invertebrates (such as bivalve mollusks, sponges, and echinoderms), it has also been discovered that they are an important source of polysaccharides or glycoconjugates, which are non-covalently linked complexes that include a polysaccharide with a protein component. Marine-derived polysaccharides are an important resource for the development of immunomodulators. For example, immunomodulatory compounds such as sulfated polysaccharides have potential application in the treatment of infections, immunodeficiencies, and cancer [13]. 

#### 4.3.1. Mollusks

Recently, an ASPG-2 polysaccharide was isolated from the mollusk *Arca subcrenata* Lischke, commonly used as a food with high nutritional value. ASPG-2 is a water-soluble glucan with a molecular weight of 4.39 × 10^5^ kDa, and it has shown significant immunomodulatory effects and no cellular toxicity. It promoted NO secretion and increased phagocytosis in murine RAW 264.7 macrophages. Its action is associated with the activation of the TLR4-MAPK/Akt-NF-κB signaling pathway. Furthermore, ASPG-2 polymerizes macrophages to type M1 [108].

In another study, it was shown that the polysaccharides extracted from the bivalve mollusk *Mytilus coruscus* (MP) had an anti-inflammatory and immunomodulatory effect on LPS-stimulated murine RAW 264.7 cells. In mice with dextran sodium sulphate-induced ulcerative colitis (DSS) after treatment with MP, the integrity of the intestinal barrier and the Firmicutes/Bacteroidetes ratio were improved; there was also a greater abundance of some probiotics, such as *Anaerotruncus*, *Lactobacillus, Desulfovibrio*, *Alistipe, Odoribacter*, and *Enterorhabdus*, in the colon. Thus, MP could be a promising dietary candidate for treating ulcerative colitis and improving immunity [109]. 

A protein with immunomodulatory activity, called HPCG2, was purified from bivalve *Scapharca broughtonii*. HPCG2 promotes the phosphorylation of Akt, ERK, and JNK. This result showed that Akt, ERK, and JNK participated in the polarization effects induced by HPCG2 on macrophages. It was associated with the regulation on TLR4/JNK/ERK and STAT3 signaling pathways in RAW 264.7 cells. This suggests that HPCG2 might be developed as a potential immunomodulatory agent or new functional product from marine organisms [110].

The oyster is a marine bivalve mollusk distributed in coastal areas and frequently used as food as for its high nutritional value [111]. In a study, they evaluated the regulating effect of oyster peptides (OPs) on the immunity of the mucosa and intestinal microflora of immunosuppressed mice, rendered such by treatment with the chemotherapy Cyclophosphamide (Cy). The immune system of the intestinal mucosa is made up of lymphocytes, macrophages, and plasma cells and is the first natural barrier against potential environmental damage [112,113]. Cyclophosphamide damages the intestinal mucosa [114] and reduces the microflora; above all, it reduces the sIgA content, which is essential for intestinal homeostasis and for the balance of the immune system [115]. An increase in the content of sIgA and of the most common bacteria in the intestinal flora, Firmicutes/Bacteriodates [116], is responsible for the production of total short-chain fatty acids SCFA (acetic acid, propionic acid, butyric acid, and acid valeric). SFCAs are metabolites that may allow the normal function of the immune system to be maintained. Moreover, it was precisely noted that, in the feces of mice treated with Cy, which were administered OP, there was an increase in SCFA, indicating an increase in intestinal bacterial activity and, therefore, an improvement in the bacterial flora [117]. The chemical investigation of mollusks has led to the isolation of a wide variety of bioactive metabolites that can be synthesized by the mollusks themselves, accumulated from food sources or produced by symbionts [118]. 

Benkendorff, determined that, as of 2014, more than 1145 compounds had been isolated from marine mollusks, including peptides, sterols, terpenes, polypropylene compounds, macrolides, fatty acid derivatives, nitrogen compounds, and alkaloids [119].

#### 4.3.2. Corals 

Corals are another source of immunomodulatory compounds. In the latter part of the last century, numerous studies were conducted to dissect the chemical composition of the main secretory product of hard (Scleractinia) and soft (Alcyonacea) corals, coral mucus, which consists of varying proportions of proteins, lipids, and polysaccharides [120,121]. The chemical composition of mucus glycoprotein differs between coral species [122]. Recently, the polysaccharide PPA was isolated from the coral *Pseudopterogorgia americana*, which showed immunomodulatory properties. It has been shown to induce pro-inflammatory mediator expression in macrophages via ROS-, MAPK-, PKC-α/δ-, and NF-κB-dependent pathways; TNF-α and IL-6 in macrophages infected with *Shigella sonnei* or *Escherichia coli*; and the phagocytosis activity of macrophages infected with bacteria [123].

#### 4.3.3. Fishes 

In a recent study, it was observed that solutions of collagen, chitosan, and collagen–chitosan extracted from fishery discards showed an immunostimulating effect on human PBMC cells. In particular, chitosan and collagen did not show cytotoxic effects. They induced the production of cytokines IL-6, IL-10, and TNF-α; the differentiation and activation of CD8+ and CD4+ T lymphocytes; and an increase in cytosolic calcium levels and of mitochondrial membrane potential [124].

In recent decades, a large number of research studies have focused on the immunomodulatory properties of host defense peptide (HPD). In particular, cationic HPD such as cathelicidin, defensin and NK-lysine (NKL) were studied [125,126,127,128]. HDPs are nothing more than antimicrobial peptides (AMPs) that, in addition to being able to kill microbes directly, they have additional immunomodulatory properties [129,130].

NKL exhibits many activities against microbial pathogens such as *Escherichia coli* and *Staphylococccus aureus* [131]; it also exhibits immunomodulatory activity [128,131,132]. So far, NKL homologous chromosomes have been identified and studied in many fish species (both from marine and freshwater environments), such as *Cynoglassus semileavis* [133], *Danio rerio* [134], *Ictalurus puntactatus* [135], *Cyprinus carpio* [136], *Oreochronis miloticus* [137], *Seophthalmus maximus* [138], and *Salmo solar* [139]; NKL expression in fish has been shown to be required to defend against invading pathogens [133,140,141,142,143,144].

Researchers have recently studied an NKL homolog, BpNKL, in *Beleophthalmus pectinirostris*. After *Edwardisiella tarda* infection, BpNKL mRNA expression was upregulated in three immune-tissues (gill, spleen, and kidney). They then investigated the effects of BpKNL on monocyte/macrophage regulation, both in vivo and in vitro. Furthermore, they compared the effects of BpNKL with commercial antibiotics (Kanamycin) against *Edwaedisiella tarda*, *Vibrio parahoemolyticus*, and *Vibrio alginolyticus*, and found that it was stronger [145]. 

Fishes also produce diverse classes of host defense peptides, such as cathelicidins, hepcidins, piscidins, pleurocidins, histone-derived, and defensine (83). Fish β-defensine has been reported to show immunomodulatory and chemotactic responses [68,146,147,148] and anti-inflammatory activity [149].

In a study of Raveendran et al., β-defensin (Lc-BD) was isolated and characters for the first time from the Asian sea bass, *Lates calcarifer*. The phylogenetic analysis of Lc-BD, showed a close relationship with β-defensins from fishes such as *Siniperca chuatsi*, *Argyrosomus regius*, *Trachinotus ovatus*, and *Oplegnathus fasciatus* [150]. Recently, a novel β -defensin, called On-Def, was studied from red-toothed triggerfish, *Odonus niger*. It was isolated from gill mRNA [151]. The most recent immunomodulatory chemical constituents isolated from marine macroorganisms that are mentioned in the text are listed in Table 2.

## 5. Discussion 

The growing demand for new drugs deriving from natural sources has pushed the field of modern biotechnologies to search for alternative sources of bioactive components with potential applications in various industrial sectors, such as the pharmacological and nutraceutical fields [4]. In recent years, marine organisms have aroused great interest in health applications in the field of immunomodulation, thanks to the plethora of high-added-value compounds isolated from them. In the last decades, a series of biologically active molecules has been extracted/isolated and purified from numerous sources of marine origin with the aid of distinct techniques and methodologies for new therapeutical applications. These compounds have been shown to have various activities, including anticancer, antiallergic, anti-inflammatory, immunomodulatory, antibacterial, and antiviral activities [9,14]. The classes of compounds that have immunomodulatory activity include polysaccharides, alkaloids, polyphenols, sterols, vitamins, proteins, peptides, different classes of lipids, and pigments [21,152]. Generally, these molecules can stimulate cells involved in the immune response such as macrophages, lymphocytes B and T, dendritic cells, and natural killer (large granular lymphocytes). There is one study on mesenchymal stem cells and a few on direct immunostimulant effects on fishes.

According to the database MarinLit (https://marinlit.rsc.org/; accessed on 20 June 2022), which is dedicated to marine natural products research, there are actually 38.795 marine compounds and about 38.497 published articles. However, there are only 14 drugs derived from marine compounds on the market, 4 in phase III clinical trials, 12 in phase II, and 7 in phase I (https://www.midwestern.edu/departments/marinepharmacology/clinical-pipeline; accessed on 20 June 2022). This testifies that the route to the market is very long and only a very small percentage of compounds reaches the market. Many marine species are still unknown, especially considering extreme environments, such as cold or deep habitats. The National Oceanic and Atmospheric Administration (NOAA) estimates that 80% of the oceans remain unexplored [1]. This increases the chance of discovering new bioactive compounds with immunomodulatory activity, trying to find alternatives to limit the costs of immunotherapies already on the market, reduce side effects, and improve activity in the treatment of human pathologies [153,154,155,156]. Many marine organisms such as microalgae, macroalgae, mollusks, and fishes are a source of macronutrients and are, in several cases, already used as food in many countries worldwide. For instance, some microalgae have received the “GRAS” status, which stands for “generally recognized as safe” [157], and it is now common to find on the market products based on microalgae-derived powder. Additional studies are also reporting their possible use as ingredients for fresh pasta, cookies, and yogurt, as recently reported by Matos et al. [158] and Ferreira et al. [152]. Various studies have also highlighted the beneficial properties of marine organisms as a food source due to the presence of compounds that stimulate the immune system [153,154]. 

Marine compounds are often used as lead compounds, but chemical synthesis and structure modification experiments have allowed researchers to improve the activity of specific compounds and the reduce side effects. For examples, the lead compound dolastatin 10 was modified, and a synthetic analogous named monomethyl auristatin E (MMAE) and monomethyl auristatin F (MMAF) became part of the new drugs brentuximab vedotin, polatuzumab vedotin, and belantamab madofotin-blmf that are currently on the market [22]. Brentuximab vedotin is an anticancer antibody–drug conjugate that comprises the anti-CD30 monoclonal antibody cAC10 conjugated to the cytotoxic agent MMAE [159]. Polatuzumab vedotin is also a conjugated antibody–drug. The CD79b monoclonal antibody is covalently cleaved to the dolastatin 10 analog, MMAE. After recognition of the B cell with the CD76b antibody, poluzumab vedotin is internalized; then MMAE enters the cell and initiates apoptosis [160]. Finally, belantamab mafodotin comprises an antibody targeting B-cell maturation antigen (BCMA) conjugated to the microtubule inhibitor MMAF. The antibody binds to BCMA on the surface of the tumor cells, and the cytotoxic microtubule inhibitor MMAF enters the cell [161].

An interesting aspect is also that the interactions between some marine species, such as algae and bacteria, that can lead to the stimulation of specific compound production or their inhibition as a defense mechanism or simply for chemical communication. For example, it is known that some mutualistic interactions between algae and bacteria, thanks to the exchange of nutrients, stimulate the algae to grow and produce high-value bioproducts. This occurs, for example, between *Chlamydomonas reinhardtii* and a heterotrophic bacterium *Mesorhizobium* sp., which produces vitamin B12, or between *Lobomonas rostrata* and *Mesorhizobium* sp. [162]. In the review by Lutzu et al., there is an exhaustive overview of the most important symbiotic partners of microalgae that are useful for biotechnological applications [163]. Other examples are the interactions between bacteria and sponges [164], and various studies suggested that bioactive compounds may be produced by both. In general, the marine biome, among numerous natural sources, appears to be an excellent source for isolating a range of biologically active constituents with medicinal values. Excellent examples to produce immunomodulatory compounds are the drugs actually on the market, namely Adcetris^®^, Polivy™, and Blenrep™, which were originally isolated from mollusk and symbiotic marine cyanobacteria (https://www.midwestern.edu/departments/marinepharmacology/clinical-pipeline; accessed on 1 June 2022) [22]. Currently, studies on new products against cancer and other diseases are often focused on immunotherapy. The uncontrolled proliferation of cancer cells leads to an accumulation of DNA mutations. These mutations are advantageous for the cancer cell, which acquires greater resistance, but, at the same time, the more that mutations accumulate, the more mutated cells are recognized and eliminated by the immune system [165,166,167].

Another interesting aspect that is not to be underestimated is the use of the omics approaches. The development of sequencing technologies; the improvement in nucleic acid extraction procedures; and the production of new molecular resources related to genome, metagenome, transcriptome, and metatranscriptome sequencing has allowed us to detect the biosynthetic pathways responsible for the synthesis of bioactive natural products. These discoveries have allowed researchers to use these key enzymes as targets for homologous or heterologous expression in order to increase the production of the metabolites of interest and implementing their possible market applications [168,169]. However, advancements have been applied, or will be applied, in order to reduce the costs and time investment required for the discovery of new products [170,171]. These approaches can help in speeding up the discovery of natural products with different potential bioactivities within different genera by identifying clusters of genes that are responsible for their synthesis [3]. An example of an in silico study has been recently published in 2022 by Widyaningrum [56], whose purpose was to study the immunomodulatory activity of microalgal pigments and propose a first clue in regard to the mechanism of microalgal pigments that modulate the human immune system. Overall, this review shows that marine organisms can produce bioactive molecules to stimulate the immune system and are worthy of further investigation.

## Figures and Tables

**Table 1:** Compounds/extracts from marine microorganisms with immunomodulatory activity. YCP stands for α-D-glucan, IL-6 for interleukin 6, IL-1 β for interleukin 1β, TNF-α for tumor necrosis factor alpha, DSS for dextran sodium sulfate, SOD for superoxide dismutase, and CAT for catalase.

Compound/Extract	Activity (Cells)	Organism	Mechanism of Action	Active Concentration	Reference
α-d-glucan	Immunomodulatory functions by regulating T lymphocytes and dendritic cells in vitro.	Marine fungus *Phoma herbarum* YS4108	After 7 days, there was relief in the clinical symptoms of mice with colitis, restoration of intestinal immune homeostasis, and remission of mucosal damage. YCP blocked the overexpression of the pro-inflammatory cytokines IL-6, TNF-α and IL-1β induced by DSS in the colon.	In vivo: 40 mg/kg Intraperitoneal injection	[24,26]
Irish deep-sea organism extracts	Induce human mesenchymal stem cell (hMSC) differentiation.	Filamentous fungi	Reduced the production of pro-inflammatory cytokines, such as TNFα and IL-1β.	In vitro: 125 µg/mL	[27]
β-carotene	Anti-inflammatory	Microalgae	Docked pro-inflammatory proteins IL-6 with binding energy −7.9 Kcal/mol.	In silico study	[48]
Phycocyanobilin	Anti-inflammatory	Microalgae	Docked pro-inflammatory protein NF-κB inducing kinase (NIK) with binding energy −9.9 Kcal/mol.	In silico study	[48]
9-cis-β-carotene	Anti-inflammatory	Microalgae	Docked pro-inflammatory protein TNF-α with binding energy −7.9 Kcal/mol.	In silico study	[48]
Exopolysaccharides (EPS)	Immunostimulatory Antibacterial	Microalga *Porphyridium cruentum*	Increase in total hemocytes (THC) value, phagocytotic activity (PA), and respiratory burst (RB).	Treatment with increasing concentration of EPS	[41]
β-glucans	Immunostimulatory, Anti-inflammatory and Antioxidant effects in fishes Promoting the growth of commensal microbiota	Microalga *Phaeodactylum tricornutum*	Elicited an inflammatory response with the downregulation of pro-inflammatory cytokines IL-1β, IL-6, and TNF-α; activated the host’s innate immune cells; increased activity of SOD and CAT in the intestine and erythrocytes in the blood; and decreased intestinal IL-1β, IL-6, and TNF-α intestinal expression.	0.6 g β-glucans per kg of feed	[54]

**Table 2:** Compounds/extracts from marine macroorganisms with immunomodulatory activity. The IL-1β stands for interleukin 1 β, IL-6 for interleukin 6, IL-10 for interleukin 10, LPS for lipopolysaccharide, TNF-α for tumor necrosis factor alpha, INF-γ for interferon gamma, MODCs for monocyte-derived dendritic cells, PBDCs for peripheral blood dendritic cells, NO for nitric oxide, ERO for endoplasmic reticulum oxidoreductin, and TGF-β for transforming growth factor beta.

Compound/Extract	Activity (Cells)	Organism	Mechanism of Action	Active Concentration	Reference
Ulvan	Immunomodulatory activity	Alga: *Ulva ohnoi*	Increases in IL-1β, IL-6, and IL-10; improves LPS-induced inflammation; and a decreases prostaglandin E2.	100 µg/mL in vitro	[76]
Fucoidan	Immunostimulatory activity Pro-proliferative activity	Algae: *Ecklonia cava*, *Macrocystis pyrifera*, *Undaria pinnatifida*, and *Fucus vesiculosus*	Increases the production of IL-6, IL-12, and TNF-α in MODCs and PBDCs; induces INF-γ production.	100 µg/mL in vitro	[85]
Sulphated polysaccharides: sulfated galactans and pyruvates (named SP1 and SP2)	Immunostimulatory activity	Alga: *Caulerpa cupressoides*	Increased production of NO, ERO, and the cytokines IL-6 and TNF-α in murine macrophages RAW 264.7.	100 µg/mL in vitro	[8]
Sulphated oligosaccharide(GLSO)	Immunomodulatory activity	Alga: *Gracilaria lemaneiformis*	Inhibits the production of INF-γ by T cells in ovalbumin (OVA) immunized mice and in vitro activation system of OVA-specific CD4^+^ T cells; inhibits the activity of mTOR, glycolysis, cell cycle, and DNA replication.		[87]
Crude extract	Immunomodulatory activity	Sponge: *Haliclona (Soestellla)* sp.	Decrease of immune cells (WBC, lymphocytes, platelets, BMC, and splenocytes) and of the splenocytic index; increase of neutrophil:lymphocyte ratio.	In vivo 15 mg/kg; 10 mg/kg; 5 mg/kg.	[97]
ThisAgeliferins derivates	Immunomodulatory activity	Sponge: *Astrosclera willeyana*	Inhibition of Cbl-b ubiquitin ligase activity (IC50 values ranging from 18 to 35 µM);	10–50 µM	[99]
ASPG-2 polysaccharide	Immunomodulatory activity	Mollusk: *Arca subcrenata* Lischke	promotes NO secretion; increases phagocytosis in murine RAW 264.7 macrophages and activation of the TLR4-MAPK/Akt-NF-κB signaling pathway.	In vitro: 250 μg/mL and 500 μg/mL for 24 h	[102]
Polysaccharides	Immunomodulatory activity Anti-inflammatory activity	Mollusk: *Mytilus coruscus*	Promotes the abundance of some probiotics in the colon.	In vitro: 300 μg/mL and 600 μg/mL	[103]
Protein HPCG2	Immunomodulatory activity	Mollusk: *Scapharca broughtonii*	Promotes the phosphorylation of Akt, ERK, and JNK.	In vitro: 250 μg/mL and 500 μg/mL	[104]
Polysaccharide PPA	Immunomodulatory activity	Coral: *Pseudopterogorgia americana*	Induces pro-inflammatory mediator expression in macrophages via ROS-, MAPK-, PKC-α/δ-, and NF-κB-dependent pathways.	In vitro: 10 μg/mL	[117]
BpNK-Lysine	Immunomodulatory activity Antibacterial activity	Fish: *Beleophthalmus pectinirostris*	In vivo, decreased the tissue bacterial burden of mudskipper infected by *E. tarda*, upregulated the mRNA expression of pro-inflammatory cytokines (IL-1β, TNF-α and IFN-γ), and downregulated the mRNA expression of anti-inflammatory cytokines (IL-10 and TGF-β) in *Beleophthalmus pectinirostris.*	In vivo: injection of 1.0 µg/g In vitro: 1.0 μg/mL	[139]

## Data Availability

Not applicable.
